# Prognostic role of preoperative albumin to globulin ratio in predicting survival of clear cell renal cell carcinoma

**DOI:** 10.1590/S1677-5538.IBJU.2018.0012

**Published:** 2018

**Authors:** Murat Yavuz Koparal, Fazlı Polat, Serhat Çetin, Ender Cem Bulut, Tevfik Sinan Sözen

**Affiliations:** 1 Department of Urology, Recep Tayyip Erdogan University Training and Research Hospital, Rize, Turkey;; 2 Department of Urology, School of Medicine, Gazi University, Ankara, Turkey;; 3 Urology Clinic, Viranşehir State Hospital, Şanliurfa, Turkey;; 4 Department of Urology, Van Training and Research Hospital, Van, Turkey

**Keywords:** Albumins, Globulins, Carcinoma, Renal Cell

## Abstract

**Purpose:**

To investigate the prognostic role of preoperative albumin/globulin ratio (AGR) in predicting disease-free survival (DFS) and overall survival (OS) in localized and locally advanced clear cell renal cell carcinoma (RCC) patients.

**Patients and Methods:**

162 patients who met the criteria specified were included in the study. The DFS and OS ratios were determined using the Kaplan-Meier method. Univariate and multivariate Cox regression analyses were performed to determine the prognostic factors affecting DFS and OS.

**Results:**

Median follow-up period was 27.5 (6-89) months. There was a statistically significant relationship between low AGR and high pathological tumor (pT) stage, presence of collecting system invasion, presence of tumor necrosis, and a high platelet count (p = 0.012, p = 0.01, p = 0.001, and p = 0.004, respectively). According to the Kaplan-Meier survival analysis, both OS and DFS were found to be significantly lower in the low AGR group (p = 0.006 and p = 0.012). In the multivariate Cox regression analysis, collecting system invasion and tumor necrosis were found to be independent prognostic factors in predicting OS and pT stage was found to be an independent prognostic factor in predicting DFS (HR: 4.08, p = 0.043; HR: 8.64, p = 0.003 and HR: 7.78, p = 0.041, respectively).

**Conclusion:**

In our study, low AGR was found to be associated with increased mortality and disease recurrence in localized and locally advanced RCC.

## INTRODUCTION

Renal cell carcinoma (RCC), the most common and lethal malignant type of kidney tumor, accounts for about 2-3% of all malignant diseases in adults. Clear cell RCC is the most common subtype of RCC, accounting for approximately 70-80% of the cases (1).

Neutrophil/lymphocyte ratio (NLR), plate-let/lymphocyte ratio (PLR), and lymphocyte/monocyte ratio (LMR) are used as markers of systemic inflammatory response. Large number of studies have reported the independent prognostic role of these markers in predicting clinical outcomes and survival of cancer patients (2–4). Albumin and globulins are major proteins that make up most all of the serum proteins. Hypoalbuminemia is not only a reflection of nutritional status in cancer patients, but is also related to systemic inflammatory response. Hyperglobulinemia is also an important marker of systemic inflammatory response and is the result of the cumulative effect of many proinflammatory cytokines involved in this pathway. Therefore, it is thought that albumin/globulin ratio (AGR), which is reflecting both the systemic inflammatory response and the systemic nutritional status, may be important prognostic factor in predicting clinical outcomes and survival of the cancer patients. In support of this knowledge, many studies in different types of cancer have demonstrated that the AGR has an independent prognostic role in predicting clinical outcomes and survival (5–9).

Our aim in this study is to investigate the prognostic role of preoperative AGR in predicting disease-free survival (DFS) and overall survival (OS) in localized and locally advanced clear cell RCC patients.

## PATIENTS AND METHODS

### Study Design

**Figure 1:**
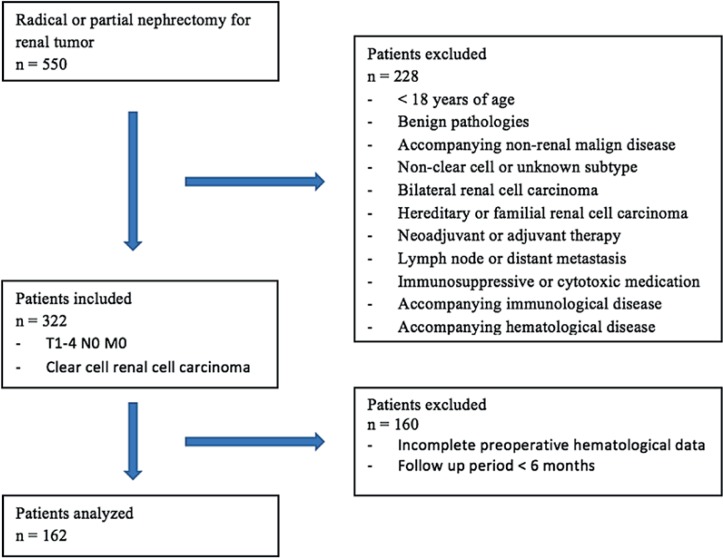
Flow chart of patients who met study inclusion / exclusion criteria.

The kidney tumor database of the Gazi University School of Medicine, Department of Urology was retrospectively screened. A total of 550 patients submitted to radical and partial nephrectomy due to renal tumor from 2010 to 2016 were identified. Patient information was collected from patient files and the hospital automation system. Of the 550 patients, 162 who met the criteria specified were included in the study (Figure-1).

### Recording Clinicopathological and Hematological Data

Clinical data, including age, sex, side, type of operation, and follow-up and pathological parameters, including pathological tumor (pT) stage, Fuhrman grade, tumor necrosis (TN), collecting system invasion (CSI), microvascular invasion (MVI), and surgical margin status, were all recorded. Patients were grouped as low grade (pT1-T2) or high grade (pT3-T4) according to Tumor-Node-Metastasis (TNM) staging (10) and grouped as low grade (grade 1-2) or as high grade (grade 3-4) according to the Fuhrman grading system (11). Since the age limit for elderly person was accepted as 65 years of age by the World Health Organization (WHO), patients were grouped as over 65 years of age and under 65 years of age.

Serum albumin and total protein levels and neutrophil, lymphocyte, platelet, and monocyte counts in the complete blood count were recorded in the hematological parameters of the patients within 15 days of operation. AGR was obtained by dividing the serum albumin value to the globulin value found by subtracting the serum albumin value from the serum total protein value. NLR was found by determining the ratio of neutrophil count to lymphocyte count, LMR was found by determining the ratio of the lymphocyte count to monocyte count and PLR was found by determining the ratio of platelet count to lymphocyte count. Patients were grouped as low or high for each parameter according to the predicted values for AGR, NLR, PLR, and LMR.

### Follow-up

Postoperative follow-up of patients was done according to tumor pathologies and stages, using chest X-ray, ultrasonography (USG), and CT at 3, 6, and 12 month intervals, depending on surgeons choice. Local recurrence or distant metastasis during follow-up was defined as disease recurrence. DFS was defined as the time from the date of operation to the date of recurrence. In patients without recurrence, DFS was defined as the time from the date of operation to the date of last follow-up visit. OS is defined as the time from the date of operation to the date of death.

#### Statistical analysis

Normal distribution of continuous variables was evaluated by visual (histogram and probability plots) and analytical (Kolmogorov-Smirnov and Shapiro-Wilk tests) methods. In the descriptive findings, categorical variables are given as numbers (percent), and continuous variables are presented with mean ± standard deviation (SD) for normal scattering data and median (minimum-maximum) for normal non-scattering data. Cut-off values for hematological data were determined using receiver operating characteristic (ROC) curve analyzes. For the categorical variables, statistical difference among groups was determined by using chi-square tests. The DFS and OS ratios were determined using the Kaplan-Meier method, and the log rank test was used to determine the statistical difference. Univariate and multivariate Cox regression analyses were performed to determine the prognostic factors affecting DFS and OS. Statistical significance was accepted as p < 0.05. IBM SPSS Statistics 15.0 was used for statistical analysis of research data.

## RESULTS

### Clinicopathological characteristics

Clinicopathological characteristics of 60 (37%) female and 102 (63%) male patients who underwent radical or partial nephrectomy followed by a localized or locally advanced clear cell RCC diagnosis are detailed in Table-1. Mean age of patients was 56.5 ± 11.8 years, and the follow-up period was median 27.5 (6-89) months. A total of 15 (9.2%) patients died during the follow-up period.

**Table 1:** Clinicopathological characteristics.

Patients (n)		162
Follow-up period (months) [median (range)]		27.5 (6-89)
Age (years) (mean ± SD)		56.5 ± 11.8
**Gender [n (%)]**		
	Female	60 (37)
	Male	102 (63)
**Side [n (%)]**		
	Right	92 (56.8)
	Left	70 (43.2)
**Type of operation [n (%)]**		
	Partial nephrectomy	60 (37.1)
	Radical nephrectomy	102 (62.9)
Diameter of tumor (cm) [median (range)]		4 (1.3-15)
**pT stage [n (%)]**		
	pT1-T2	127 (78.4)
	pT3-T4	35 (21.6)
**Fuhrman grade [n (%)]**		
	1-2	101 (62.3)
	3-4	61 (37.7)
**Surgical margin [n (%)]**		
	Positive	13 (8)
	Negative	149 (92)
**Collecting system invasion [n (%)]**		
	Yes	18 (11.1)
	No	144 (88.9)
**Microvascular invasion [n (%)]**		
	Yes	11 (93.2)
	No	151 (93.2)
**Tumor necrosis [n (%)]**		
	Yes	31 (19.1)
	No	131 (80.9)
Albumin (mean ± SD) (g / dL)		4.31 ± 0.39
Total protein (mean ± SD) (g / dL)		7.35 ± 0.49
Neutrophil [median (range)] / µL		4685 (2180-13600)
Lymphocyte [median (range)] / µL		1835 (300-4500)
Monocyte [median (range)] / µL		518 (100-1331)
Platelet (mean ± SD) / µL		257321 ± 75533
Albumin / globulin ratio (mean ± SD)		1.45 ± 0.26
Neutrophil / Lymphocyte ratio [median (range)]		2.51 (0.69-36.3)
Platelet / Lymphocyte ratio [median (range)]		132.8 (45.5-449.5)
Lymphocyte / monocyte ratio [median (range)]		3.5 (0.3-11)

**SD** = Standard deviation

### Optimal cut-off values for AGR, NLR, PLR, and LMR

**Figure 2:**
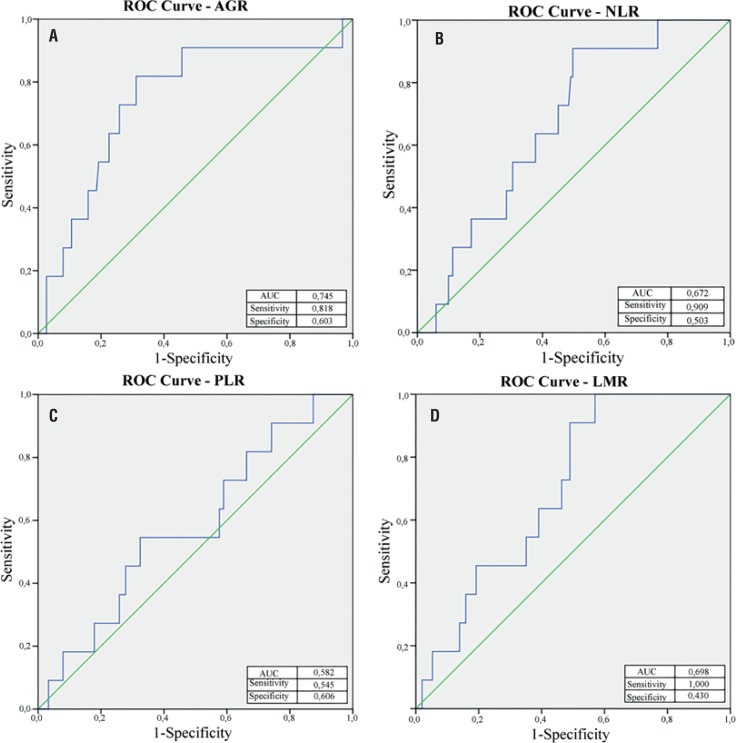
Optimal cut-off levels for (A) albumin / globulin ratio (AGR), (B) neutrophil / lymphocyte ratio, (c) platelet / lymphocyte ratio (PLR), and (D) lymphocyte /monocyte ratio(LMR) were applied with receiver operating characteristic (ROC) curves for overall survival.

**Figure 3:**
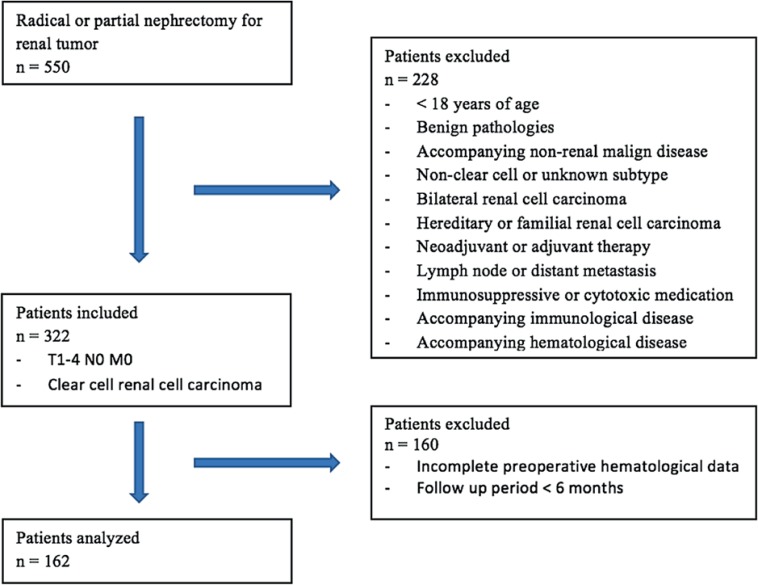
Optimal cut-off levels for (A) albumin / globulin ratio (AGR), (B) neutrophil / lymphocyte ratio, (c) platelet / lymphocyte ratio (PLR), and (D) lymphocyte / monocyte ratio(LMR) were applied with receiver operating characteristic (ROC) curves for disease-free survival.

Cut-off values of AGR, NLR, PLR, and LMR were determined at points with optimal specificity and sensitivity by ROC curves generated using DFS and OS data (Figures 2 and 3). Optimal cut-off values were determined as 1.40 (< 1.40, n = 69 and ≥ 1.40, n=93) for AGR, 2.45 (≤ 2.45, n = 77 and > 2.45, n = 85) for NLR, 148 (≤ 148, n = 101 and > 148, n = 61) for PLR, and 4 (< 4, n = 97 and ≥ 4, n = 65) for LMR. Patients were separated into two groups according to the values of these parameters as the low value group and the high value group.

### Relationships between AGR and clinicopathological characteristics

Relationships between AGR and clinicopathological characteristics are detailed in Table-2. There was a statistically significant relationship between low AGR and high pT stage, presence of CSI, presence of TN, and a high platelet count (p = 0.012, p = 0.01, p = 0.001, and p = 0.004, respectively).

**Table 2:** Associations between AGR and clinicopathological characteristics.

		Low AGR	High AGR	p value
**Age**				
	≤ 65	48 (69.6)	76 (81.7)	0.091
	> 65	21 (30.4)	17 (18.3)	
**Gender**				
	Female	28 (40.6)	32 (34.4)	0.051
	Male	41 (59.4)	61 (65.6)	
**pT stage**				
	pT1-T2	51 (73.9)	83 (89.2)	0.012
	pT3-T4	18 (26.1)	10 (10.8)	
**Fuhrman grade**				
	1-2	37 (53.6)	64 (68.8)	0.052
	3-4	32 (46.4)	29 (31.2)	
**Collecting system invasion**				
	Yes	13 (18.8)	5 (5.4)	0.01
	No	56 (81.2)	88 (94.6)	
**Microvascular invasion**				
	Yes	6 (8.7)	5 (5.4)	0.531
	No	63 (91.3)	88 (94.6)	
**Tumor necrosis**				
	Yes	22 (31.9)	9 (9.7)	0.001
	No	47 (68.1)	84 (90.3)	
Albumin (mean ± SD) (g / dL)		4.09 ± 0.38	4.48 ± 0.30	< 0.001
Total protein (mean ± SD) (g / dL)		7.49 ± 0.54	7.25 ± 0.43	0.002
Neutrophil [median (range)] / μL		4880 (2180-13600)	4430 (2560-10900)	0.145
Lymphocyte [median (range)] / μL		1720 (770-3300)	1890 (300-4500)	0.432
Monocyte [median (range)] / μL		520 (100-1331)	517 (220-1260)	0.836
Platelet (mean ± SD) / μL		277037 ± 84525	242693 ± 64773	0.004

**AGR** = Albumin / globulin ratio; **SD** = Standard deviation

### Relationships between DFS and AGR, NLR, PLR, and LMR

**Figure 4:**
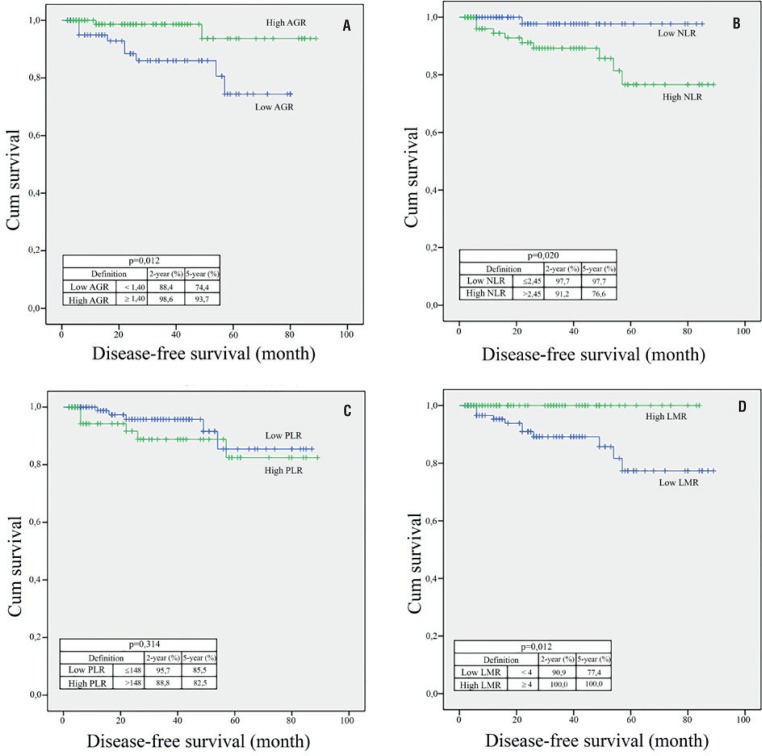
Kaplan–Meier curves predicting disease-free survival, and 2 and 5-year disease-free survival probability, groups categorized by the pretreatment (A) albumin / globulin ratio (AGR), (B) neutrophil / lymphocyte ratio (NLR), (c) platelet / lymphocyte ratio (PLR), and (D) lymphocyte / monocyte ratio (LMR)..

Median follow-up period was 27.5 (6-89) months. In 11 (6.7%) patients, disease recurrence was observed. Local recurrence was observed in two patients, and distant metastasis was seen in nine patients. According to the Kaplan-Meier survival analysis, DFS was found to be significantly lower in the low AGR group, the high NLR group, and the low LMR group (p = 0.012, p = 0.02, and p = 0.006, respectively) (Figure-4).

Table-3 shows the univariate and multivariate Cox regression analyses in terms of DFS. Thus, in univariate analyses, high pT stage, low AGR, presence of CSI, presence of MVI, and presence of TN were associated with an increased risk of disease recurrence (p = 0.026, p < 0.001, p < 0.001; p < 0.001, and p = 0.001, respectively). In the multivariate Cox regression analyses, pT stage was found to be an independent prognostic factor in predicting DFS (HR: 7.78, p = 0.041).

**Table 3:** Univariate and multivariate Cox regression analyses of prognostic factors for disease-free survival.

		Univariate analysis			Multivariate analysis		
		CI 95%	HR	p value	CI 95%	HR	p value
**Age**							
	≤ 65	0.30-4.28	1.13 1.00 (ref.)	0.852			
	> 65			
**Gender**							
	Female	0.56-8.33	2.17 1.00 (ref.)	0.259			
	Male			
**pT stage**							
	pT1-T2	4.81-69.76	18.3 1.00 (ref.)	< 0.001	1.08-55.7	7.78 1.00 (ref.)	0.041
	pT3-T4						
**Fuhrman grade**							
	1-2	0.73-7.98	2.42 1.00 (ref.)	0.144			
	3-4			
**Surgical margin**							
	Positive	0.13-8.58	1.09 1.00 (ref.)	0.933			
	Negative			
**Collecting system invasion**							
	Yes	2.64-28.52	8.68 1.00 (ref.)	< 0.001	0.72-17.82	3.6 1.00 (ref.)	0.116
	No						
**Microvascular invasion**							
	Yes	3.48-67.51	15.32 1.00 (ref.)	< 0.001	0.72-48.48	5.92 1.00 (ref.)	0.097
	No						
**Tumor necrosis**							
	Yes	2.19-23.92	7.24 1.00 (ref.)	0.001	0.84-25.13	4.6 1.00 (ref.)	0.078
	No						
**Albumin / globulin ratio**							
	≤ 1.40	1.23-26.55	5.72 1.00 (ref.)	0.026	0.36-15.10	2.34 1.00 (ref.)	0.372
	> 1.40						
**Neutrophil / Lymphocyte ratio**							
	≤ 2.45	0.01-1.00	7.79 1.00 (ref.)	0.051			
	> 2.45			
**Platelet / Lymphocyte ratio**							
	≤ 148	0.55-6.03	1.82 1.00 (ref.)	0.323			
	> 148			
**Lymphocyte / monocyte ratio**							
	< 4	0.24-7080	4.18 1.00 (ref.)	0.154			
	≥ 4						-

**CI** = Confidence interval; **HR** = Hazard ratio; **Ref** = Reference

### Relationships between OS and AGR, NLR, PLR, and LMR

**Figure 5:**
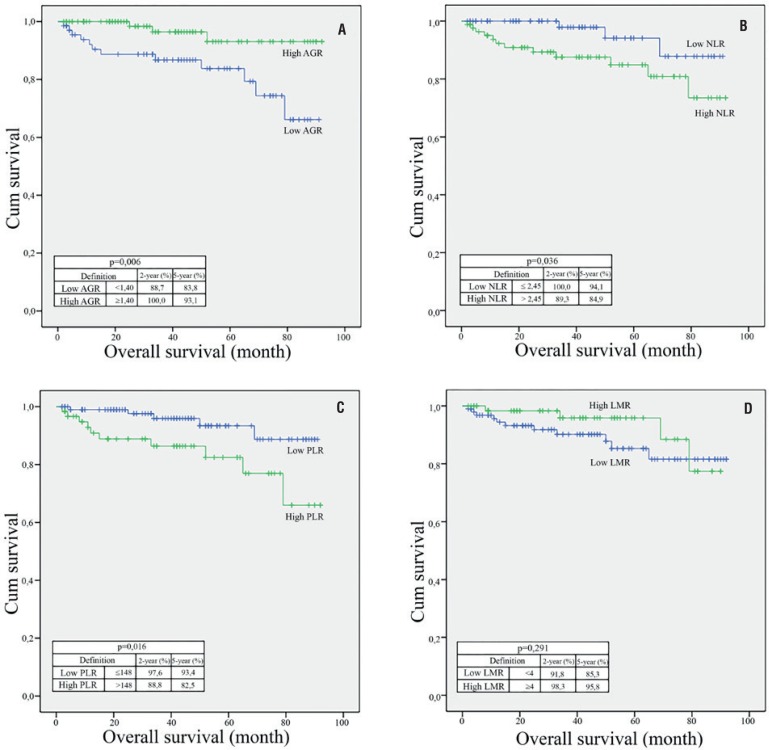
kaplan–Meier curves predicting overall survival, and 2 and 5-year overall survival probability, groups categorized by the pretreatment (A) albumin / globulin ratio (AGR), (B) neutrophil / lymphocyte ratio (NLR), (c) platelet / lymphocyte ratio (PLR), and (D) lymphocyte / monocyte ratio (LMR).

Median follow-up was 27.5 (6-89) months and 15 (9.2%) patients died during the follow-up period. According to Kaplan-Meier survival analysis, OS was found to be significantly lower in patients in the low AGR group, high NLR group, and high PLR group (p = 0.006, p = 0.036, and p = 0.016, respectively) (Figure-5).

Table-4 details the univariate and multivariate Cox regression analyses in terms of OS. Thus, in univariate analyzes, low AGR, high NLR, high PLR, high pT stage, presence of CSI, presence of MVI, and presence of TN were associated with an increased risk of death (p = 0.014, p = 0.049, p = 0.024, p = 0.005, p = 0.003, and p < 0.001, respectively). In the multivariate Cox regression analysis, CSI and TN were found to be independent prognostic factors in predicting OS (HR: 4.08, p = 0.043 and HR: 8.64, p = 0.003, respectively).

**Table 4:** Univariate and multivariate cox regression analyses of prognostic factors for overall survival.

		Univariate analysis			Multivariate analysis		
		CI %95	HR	p value	CI %95	HR	p value
**Age**							
	≤65	0.74-5.88	1.00 (ref.) 2.09	0.162			
	>65			
**Gender**							
	Female	0.67-6.74	2.12 1.00 (ref.)	0.199			
	Male						
**pT stage**							
	pT1-T2	1.57-12.7	4.48 1.00 (ref.)	0.005	0.22-5.40	0.56 1.00 (ref.)	0.452
	pT3-T4						
**Fuhrman grade**							
	1-2	0.80-6.14	2.21 1.00 (ref.)	0.126			
	3-4						
**Surgical margin**							
	Positive	0-116	0.043 1.00 (ref.)	0.436			
	Negative						
**Collecting system invasion**							
	Yes	1.70-13.54	4.81 1.00 (ref.)	0.003	1.03-8.63	4.08 1.00 (ref.)	0.043
	No						
**Microvascular invasion**							
	Yes	1.87-27.42	7.17 1.00 (ref.)	0.004	0.63-14.4	2.14 1.00 (ref.)	0.143
	No						
**Tumor necrosis**							
	Yes	2.21-17.07	6.15 1.00 (ref.)	< 0.001	1.68-13.42	8.64 1.00 (ref.)	0.003
	No						
**Albumin / globulin ratio**							
	≤ 1.40	0.57-0.72	4.92 1.00 (ref.)	0.014	0.10-1.61	1.38 1.00 (ref.)	0.239
	> 1.40						
**Neutrophil / Lymphocyte ratio**							
	≤ 2.45	1.00-12.6	1.00 (ref.) 3.56	0.049	0.31-6.26	1.00 (ref.) 0.58	0.443
	> 2.45						
**Platelet / Lymphocyte ratio**							
	≤ 148	1.17-10.08	1.00 (ref.) 3.44	0.024	0.87-7.74	1.00 (ref.) 2.97	0.084
	> 148						
**Lymphocyte / monocyte ratio**							
	< 4	0.58-5.76	1.83 1.00 (ref.)	0.298			
	≥ 4			

## DISCUSSION

A better understanding of cancer biology has shown that increased systemic inflammatory response and impaired systemic nutritional status are associated with poor prognosis (12).

AGR, obtained by dividing the serum albumin level by the globulin level, is a valuable indicator that reflects both systemic nutritional and systemic inflammatory status in cancer patients with a single measurement. In our study, we determined the optimal cut-off value for AGR as 1.45 in the ROC curve analysis, and we create two groups as low AGR and high AGR. In our study, we found that preoperative low AGR was statistically associated with the pT3-T4 stage, presence of CSI, and presence of TN (p < 0.05). The Kaplan-Meier survival analysis for DFS and OS assessments revealed that recurrence and mortality rates were higher in patients in the low AGR group (p = 0.012 and p = 0.006, respectively). In the multivariate Cox regression analysis, we found that low AGR was associated with increased risk of recurrence by 2.34 times and increased risk of mortality by 1.38 times, but we could not statistically assess the independent prognostic role of AGR in predicting disease recurrence and risk of death (p > 0.05). There are a limited number of studies in RCC that explore the prognostic role of AGR in terms of survival. He et al. (5), in their retrospective evaluation of 895 cases of RCC from all stages, determined the cut-off value of AGR as 1.47. As a result of the multiple variable Cox regression analysis they performed, they found that low AGR was associated with increased mortality and AGR was an independent prognostic factor in predicting OS (HR: 0.63, 95% CI [0.43-0.93], p = 0.022). In a study by Chen et al. (6), they determined a cut-off value of AGR as 1.22 in their retrospective evaluation of localized and locally advanced 416 clear cell RCC cases, and they showed that AGR has an independent prognostic role in predicting both OS (HR: 6.79, 95% CI [3.21-14.37], p < 0.001) and DFS (HR: 8.80, 95% CI [3.89-19.92], p < 0.001). In this study, NLR and LMR were also found as independent prognostic factors in predicting DFS and OS, and PLR was shown to have no independent prognostic role in predicting DFS and OS.

In a large number of studies, it has been shown that preoperative high NLR, low PLR, and low LMR are associated with a poor prognosis in many types of cancers, including urinary tract cancers (2, 3, 13). In our study, the cut-off values for NLR, PLR, and LMR were 2.45, 148 and 4, respectively. Kaplan-Meier survival analysis showed significantly higher recurrence and mortality rates in patients in the high NLR group (p = 0.02 and p = 0.036, respectively). We found that the mortality rates in patients in the high PLR group and recurrence rates in patients in the low LMR group were significantly higher (p = 0.016 and 0.006, respectively). In the multivariate Cox regression analysis, we found that NLR, PLR, and LMR had no independent prognostic role in predicting DFS and OS. In a meta-analysis by Boissier et al. (14) that evaluated the prognostic role of NLR in RCC, it was shown that NLR > 3 was associated with recurrence of disease in localized RCC and that NLR has an independent prognostic role in predicting DFS (HR: 1.63, 95% CI [1.15-2.29]), but not in predicting OS. In locally advanced and metastatic RCC, it was shown that NLR has an independent prognostic role in predicting both progression (HR: 3.19, 95% CI [2.23-4.57]) and mortality (HR: 1.55 [1.36-1.76]). Estimates ranging from 2.5 to 5 for NLR were used in the studies that were evaluated in this meta-analysis. Hutterer et al. (15) showed the independent prognostic role of LMR (cut-off value 3) in predicting local and locally advanced clear cell RCC in their study (HR: 1.55, CI 95% [1.10-4.94]). Lucca et al. (16). used MLR instead of LMR and demonstrated that high MLR was associated with higher risk of disease recurrence and MLR has an independent prognostic role in predicting DFS (HR: 5.78, 95% CI [1.78-18.73]). Results have been published showing that PLR may have an independent prognostic role in predicting survival and treatment response in metastatic disease. No results supporting the independent prognostic role in predicting survival in local and locally advanced RCC have been reported (17, 18).

In renal cell carcinoma, pT stage is one of the most important prognostic factors (19). According to the multivariate Cox regression analysis in our study, we found the pT stage is an independent prognostic factor in predicting DFS. The presence of TN has been shown to be associated with a poor prognosis in many studies and has been considered as a poor prognostic factor by the WHO / International Society of Urological Pathology (WHO/ISUP) (20). As a result of the multivariate Cox regression analysis in this study, we found that TN was as an independent prognostic factor in predicting OS in accordance with the literature. In the TNM grading system for renal tumors updated by AJCC, the CSI was not included in the previous grading system, but now it is evaluated as T3a in the updated version. In support of this update in our study, we have found CSI to be an independent prognostic factor in predicting OS. The Fuhrman grading system, which has been shown to be an important prognostic factor in RCC, now, however, requires modification due to its inadequacy in evaluation, and, currently, the use of the WHO / ISUP rating system is recommended (21). In our study, we did not find the association of the Fuhrman degree with DFS and OS as a result of the univariate and multivariate Cox regression analyses (p > 0.05). Although we showed that MVI, which is controversial as to its prognostic role of RCC (20), has an association with OS and DFS in univariate analysis, we determined that it is not an independent prognostic factor as a result of the multivariate Cox regression analysis. We also could not show the relationship of age, gender, and surgical marginal status that we had considered as possible prognostic factors with DFS and OS (p > 0.05).

## CONCLUSIONS

In this study, we found that DFS and OS were significantly lower in patients within the low AGR group. We also found TN and CSI in predicting OS, and pT stage in predicting DFS, as independent prognostic factors. The retrospective nature of the study, the limited number of patients, and the short follow-up period of patients are the most significant limiting factors. Since markers, such as AGR, NLR, PLR, and LMR, are not disease-specific markers, prospective studies should be carried out with multi-center by creating nomograms specific to RCC in order to increase the prognostic value of these markers.

## ABBREVIATIONS

RCC =: Renal cell carcinoma
NLR =: Neutrophil / lymphocyte ratio
PLR =: Platelet / lymphocyte ratio
LMR =: Lymphocyte / monocyte ratio
AGR =: Albumin / globulin ratio
DFS =: Disease-free survival
OS =: Overall survival
TN =: Tumor necrosis
pT =: Pathological tumor
CSI =: Collecting system invasion
MVI =: Microvascular invasion
TNM =: Tumor-Node-Metastasis
WHO =: World Health Organization
SD =: Standard deviation
ROC =: Receiver operating characteristic
HR =: Hazard ratio
CI =: Confidence interval
MLR =: Monocyte / lymphocyte ratio
ISUP =: International Society of Urological Pathology
AJCC =: American Joint Committee on Cancer
Ref =: Reference
AUC =: Area under curve
